# Psychological processes and abilities for ceasing sex as self-injury– a qualitative study

**DOI:** 10.1186/s12888-025-07029-2

**Published:** 2025-07-01

**Authors:** Cathrine Apelqvist, Tove Irmelid, Linda S. Jonsson, Cecilia Fredlund

**Affiliations:** 1 Department of Psychology, University of Gothenburg, Gothenburg, Sweden; 2 Department of Social Sciences, Marie Cederschiöld University, Stockholm, Sweden; 3 Department of Psychiatry in Linköping, Department of Biomedical and Clinical Sciences, Barnafrid- Swedish National Center on Violence Against Children, Linköping University, Linköping, Sweden

**Keywords:** Sex as self-injury, Nonsuicidal self-injury, Sexual violence, Sexual abuse, Psychological interventions

## Abstract

**Background:**

The term sex as self-injury (SASI) refers to sexual behaviors that are used as a means of self-injury, with motives such as emotional regulation comparable to other self-injurious behaviors, including burning or cutting the skin. The aim of this study was to explore which psychological processes and abilities that made it possible to cease SASI, to contribute to the knowledge that underpins psychological interventions and treatments.

**Method:**

The study was based on an open-ended questionnaire published on the websites of Swedish NGOs offering help and support to women and youths. In total 196 individuals with experience of SASI were included in the study. The age of the participants was 15–64 years (mean age 27.9 years), and most of the participants were women. Thematic analysis was used for the study, with the preunderstunding of cognitive behavioral therapy treatment and functional analysis.

**Results:**

Five abilities were seen as important for cessation of SASI; (1) *Revised core beliefs about the self* which were achieved through new experiences or cognitive restructuring. (2) *Evolved emotional competence* achieved through understanding or acceptance of emotions or new coping skills. (3) *Increased relational competence* via new relationship experiences or new communication skills. (4) *Acquired meta-perspective* through insight and knowledge of SASI. (5) *Strengthened psychological empowerment* through new relationships to the body and sexuality, transfer of responsibility or norm-critical perspective.

**Conclusions:**

Based on the results regarding psychological processes, acquired abilities and alternative behaviors, proposals for therapeutic interventions that may activate these processes were discussed.

**Supplementary Information:**

The online version contains supplementary material available at 10.1186/s12888-025-07029-2.

## Background

The urge to live and survive is strong in humanity. Despite this, it has been described since time immemorial how people engage in behaviors that threaten their existence. Some of these behaviors can be downright lethal, such as intentionally attempting to take one’s own life. There are also behaviors that intend to cause damage to one’s own body tissue, without the intention of ending life but to fulfill other functions [16]. Historically, different concepts for this phenomenon have been used, such as self-injurious behavior, deliberate self-harm, self-mutilation and nonsuicidal self-injury (NSSI). NSSI, the term that will be used in this study, has in the latest edition of the DSM been suggested as a diagnosis that entails deliberate damage to one’s own body tissue in the absence of suicidal intention and for purposes not socially sanctioned, used as a means of obtaining relief from negative feelings or cognitive state, resolving interpersonal difficulties or inducing positive feelings [2]. In research a distinction is made between direct and indirect self-injurious behaviors where, for example, sexual risk behavior is of the latter. Indirect self-injury is rarely included in the definition of NSSI [5, 17]. In recent years, studies have found that other behaviors such as sexual activities could be used with the intention of inflicting harm to oneself with the same purpose and function as NSSI despite differences in topography [8, 10, 13]. The research concerning sex as self-injury (SASI) is still limited, and to our knowledge no earlier study has investigated the psychological processes and abilities required to cease SASI.

### Sex as self-injury

There are few epidemiological studies investigating the prevalence of SASI. In a study of Swedish high-school seniors, mean age 18 years, the prevalence of SASI was found to be 2.2%, more commonly seen among girls than boys [7]. In a small pilot study of 50 US college students, 12% reported having used sex as a means of self-injury [14]. In many respects, SASI and NSSI could be seen as functionally equivalent behaviors, even though the topography differs, by sharing the most common functions such as emotional regulation including reducing feelings of being numb and empty, stopping negative emotions or feeling something even if it is pain. In SASI, socially related functions are more common than in NSSI, such as getting attention or getting a reaction from someone, even if it is a negative one [10, 20]. The context of SASI could be very diverse, including sexual contacts where sexual or physical violence is expected. It could include sex with a person without feeling attraction or even feelings of aversion, such as having sex with a man even though the person is attracted to women as a means of punishing or injuring oneself. It could be allowing others to do what they want with one’s body despite the lack of lust or desire. Other examples may include constantly seeking out new sexual contacts or repeating previous sexual abuse. Most often, SASI occurs with one or more sexual partners, but it has been described in situations of masturbation by injuring one’s genitalia, in sexual contacts through the internet, but also in the framework of relationships [8].

SASI has been associated with sexual abuse, especially penetrative sexual abuse, early sex debut, experience of selling sex, lower use of contraception, experience of more sexual partners and higher abortion rates. In comparison with NSSI, SASI is more associated with earlier experiences of sexual abuse and trauma symptoms such as post-traumatic stress, dissociation and sexual problems [7, 23]. Qualitative research has described poor mental health, anxiety, self-loathing and re-experiencing as part of PTSD as reasons for SASI being continued [8].

### Sex as self-injury and revictimisation

The phenomenon of revictimisation describes how exposure to sexual abuse during childhood often entails increased risks of being sexually exposed again later in life. A meta-analysis [19] of risk factors for revictimization after childhood sexual abuse found that sexual risk-taking, especially during adolescence, is a link that may explain the connection between past abuse and later revictimization. In parallel with sexual risk-taking, post-traumatic stress, emotional dysregulation and maladaptive coping strategies emerge as additional mediating factors [19]. Using sex as a strategy to reduce negative emotions was found as a risk factor for sexual revictimization. The explanation may be that sexual abuse during upbringing creates psychological suffering, for example in the form of PTSD. The victim may develop problematic coping strategies to regulate difficult emotions, for example by exposing themselves to sexual risk-taking such as having sex with many different and/or unknown partners [18]. Research on SASI discusses the possibility that the same connection could be relevant when it comes to sex as self-injury. Earlier studies have found that SASI was often preceded by sexual abuse that had created emotional dysregulation, where a harmful way of using sex becomes a strategy for emotion regulation but at the same time entails negative consequences and risks of continued vulnerability [7, 8]. A complex connection between SASI and sexual violence has been found where earlier experience of sexual abuse might normalize sexual violence and lead to SASI. SASI could be used to regain control of re-experiences, the body, sexuality, and shame after sexual abuse. Further, SASI could escalate into sexual violence through an increased need for emotional regulation and increased risk-taking. SASI increases the risk of victimization and might even be viewed as part of sexual violence [9].

### Ceasing self-injury

Earlier research has found that one important reason for SASI to continue, despite the negative consequences, is that it is hard to stop. The relief of negative feelings during the sexual encounter and the even stronger painful emotions afterwards create a vicious circle. Increased feelings of guilt, shame and self-contempt after SASI trigger the behavior to start all over again due to the underlying function of emotional regulation of the behavior [8]. In a study of perceived help and support in relation to SASI, informants described the importance of having a word for the behavior, and to receive questions and information about the function of the behavior. In the same study, flexible, respectful and professional help and support from an early age was described as important, as well as help with the underlying reasons for SASI, in the form of therapy for traumatic events, PTSD and anxiety, for example [6]. SASI has also been associated with higher frequency of contact with healthcare due to depression, anxiety, eating disorders, ADHD/ADD, autism, suicide attempts, alcohol and drug use and might be healthconcerns that could be helpful to address in relation to SASI [7].

A meta-analysis of research on ceasing self-injurious behaviors shows that both inter- and intra-personal factors affect opportunities and incentives to stop self-injury and that these can be categorized into themes such as: social support, self-esteem, emotional regulation, personal motivation and professional support [15]. When it comes to incentives for NSSI, a distinction is made between intrapersonal reasons, such as affect regulation and self-punishment, and interpersonal reasons, such as a desire to influence others or create a group belonging. It appears that intrapersonal reasons more clearly maintain NSSI, making it harder to stop [12]. Different reasons for ceasing self-injury have been demonstrated, where aspects such as desire for change, reduced emotional load and concern about negative consequences of NSSI are associated with greater resilience and protection [21]. These are aspects that are linked to hopefulness and a belief that it is possible to stop hurting oneself, as well as to social support, values in life and adaptive coping [21]. Social support, improved quality of life and improved ability to regulate emotions are essential and conducive to ceasing self-injury [11].

Young people with SASI are a vulnerable group at risk of further sexual revictimization and traumatization [9]. Existing research on SASI examines prevalence, comorbidity, function and supporting explanations for the behavior, but there is currently a lack of research concerning the processes or abilities that lead to ceasing SASI. Increased knowledge concerning important factors for ceasing SASI could have clinical implications for therapeutic interventions for the target group. Hence, the knowledge of psychological processes and abilities that lead to cessation of SASI could contribute to the development of preventive or therapeutic interventions for SASI.

### Aim of the study

The aim of this study was to explore which psychological processes and abilities made it possible to cease SASI and thus contribute knowledge that might inform therapeutic interventions. Learning about the experiences of individuals with ongoing or previous experiences of SASI allowed the following questions to be explored:

1. What abilities are described as contributing to breaking the maintaining cycle of sex as self-injury?
2. What psychological processes are described as having developed these abilities?
3. What new behaviors can replace or serve as alternative behaviors to SASI?

## Method

### Participants and procedure

The study used a qualitative design and was based on an anonymous open-ended questionnaire that was published on the websites of 37 Swedish NGOs offering help and support for women and youths, such as women’s shelters. The collection of responses took place between December 2016 and April 2017 and included informants from all parts of Sweden. The organizations were selected based on relevance to the theme and on the basis that their websites were assumed to be visited by the target group, based on their work to offer help and support to vulnerable women and focus on young people’s mental and sexual health. The inclusion criteria were a minimum age of 15 years, and that the person had experience of SASI. Participation was anonymous, voluntary and could be terminated at any time. SASI was defined in the questionnaire as: “To have repeatedly sought sexual situations that have caused you physical and/or mental harm and that have affected you in your life.”

A total of 199 participants completed the survey, but three were excluded from the study due to lack of responses that were relevant to the study, leaving 196 informants for the study. In total 187 informants identified as woman, four as men, four as non-binary and one person did not answer the question regarding gender. The age of the participants was 15–64 years, with a mean age of 27.9. The majority (82.9%) of the informants stated that they had their first experiences of SASI during their adolescence. The study included both informants that had stopped using SASI and those with ongoing SASI.

### The questionnaire

The questionnaire used for the study consisted of 12 open-ended questions that were formulated with the aim of investigating and increasing the understanding of SASI (see Appendix). The questions concerned background information, coping strategies, motives and manifestations of SASI, and experience of help and support. This questionnaire has been used in earlier studies, but with other aims and research questions [6, 8, 9]. The questionnaire was tested in a pilot study that included five informants, leading to some changes in the structure of the questionnaire, such as more precise follow-up questions. Most participants answerd all questions but the answers were varying from one sentence to one page. Overall, the material was perceived as trustworthy since the answers were found to be genuine and well described as seen in the citations.

### Data analysis

Thematic Analysis (TA) according to Braun & Clarke’s model [3] was used for analyzing the data with the aim of identifying psychological processes and abilities to cease SASI. The six steps according to Braun & Clarke [3] were followed, including the first reading of the text, giving initial ideas and thoughts that were recorded. This was followed by further reading which resulted in suggested codes and sub-codes. The coding was first made separately by the authors CA and TI and was then discussed and compared. The codes were compiled in a codebook with a written definition. The material was then re-coded individually by CA and TI. After that, the material that the authors linked to the established codes was re-compared. Themes and subthemes were identified and discussed in the research group and resulted in the final five main themes and twelve subthemes. The study was made in Swedish but quotations were translated into English by the authors and further reviewed by professional translators. Triangulation was performed by separately coding the text that was further compared. There was continuous discussion in the research group concerning the final themes and findings. A logbook was used in which all steps and decisions in the process were recorded. The computer program Nvivo 20.7.1 was used to organize and code the survey responses.

### Pre-understanding and approach in analysis

Two of the authors (CA and TI) are social workers in training to become psychotherapists with a cognitive behavioral therapy (CBT) focus and have several years of professional experience in conducting individual therapy with, among others, clients who have had different types of self-injurious behaviors. The author CF works as psychiatrist, working clinically with patients with history of traumatization and self-injury, she also works as a researcher in the field of the subject. LJ is a social worker and a researcher in the field. Theoretical knowledge based on CBT and functional analysis was an important preunderstanding that has influenced the process of analysis. Clinical experiences have created recognition, for example, of common patterns of self-injury perceived in the data material and influenced discussions and reasoning during the analysis phase.

Most interventions in CBT treatment are based on a model called functional analysis, which means the following steps are identified for the behavior: (1) Triggers; (2) Thoughts, feelings and physical sensations; (3) Impulse to action and actions; (4) Immediate consequence of the behavior; (5) Long-term consequence of the behavior. What constitutes the function of a behavior is the immediate consequence which causes that behavior to be repeated. Problematic behaviors are characterized by the fact that there is a dissonance between the immediate and the long-term consequence, where the latter does not serve the individual’s goals. Based on a functional analysis, it is possible to determine what reinforces and maintains a problem behavior (e.g. self-injury), by defining the short-term consequence and identifying alternative behaviors that are functional over the long term. A functional understanding of behaviors has been the basis for the authors’ approach in the analysis of the study and the theoretical idea that all behaviors arise and continue because they fulfill a certain function that the individual’s inner logic renders comprehensible. Even destructive or seemingly incomprehensible behaviors can be understood from this perspective, which becomes fundamental when self-injury is viewed from a CBT perspective.

Thematic Analysis (TA) according to Braun & Clarke [3] was chosen in the analytic method, however, during the process of analysis it was clear that also other analythic methods could have been suitable especially related to the interest of investigating the process of leaving the behavior. TA was chosen since it is a flexible method in many aspects such as inductive-deductive, semantic-latent descriptive-interpretative approach. It is easy to understand and follow also for a person with less experience of qualitative research. We also aimed to find clear themes/subthemes that could be helpful in clinical work, for example in therapy, why we found the method suitable.

## Results and analysis

The main findings for the study were the five abilities; (1) Revised core beliefs about the self, (2) Increased relational competence, (3) Evolved emotional competence, (4) Acquired meta-perspective, (5) Strengthened psychological empowerment. Twelve psychological processes enabling the abilities and assisting in ceasing SASI and further alternative behaviors were described (see Table 1).

**Table 1:** Acquired abilities and processes for ceasing sex as self-injury

Acquired abilities that break maintenance loop	The process– how to get there	Examples of new behaviors after acquired ability
**Revised core beliefs about the self**	Via new experiences	*“I started to give myself an intrinsic value. I began to love myself. Then I stopped offending myself through others"* (Woman, aged 26, No. 84)
	Via cognitive restructuring	
**Evolved emotional competence**	Via understanding emotions	*“Try to accept that the feelings are there but at the same time make sure that they don’t get the better of you. Thinking about breathing. I take care of myself by taking care of basic needs such as sleep*,* food*,* hygiene*,* etc.”* (Woman, aged 20, No. 58)
	Via acceptance of emotions	
	Via coping skills	
**Increased relational competence**	Via new relationship experiences	*“Talk to my husband*,* be with my son*,* talk to a person who I think can help”* (Woman, aged 29, No. 51)
	Via new communication skills	
**Acquired meta-perspective**	Via insight and knowledge of SASI	*“I went to CBT therapy because I self-harmed with razor blades*,* and I used the same way when I stopped hurting myself with sex*,* one learns good tricks there”* (Woman, aged 25, No. 108)
**Strengthened psychological empowerment**	Via a new relationship to one’s body and sexuality	*“I write a list of everything I feel guilty about and go through them and do my best to convince myself that none of them are my fault”* (Woman, aged 15, No. 177) *“Talk to the mirror and pretend I’m speaking up”* (Woman, aged 26, No. 165)
	Via self-assertive behaviors	
	Via transfer of responsibility	
	Via a norm-critical perspective	

### Revised core beliefs about the self

A core belief about the self is an individual’s subjective truth about the self. In different ways, the informants expressed that during the time of SASI, they had low thoughts about their self-worth. This negative self-image was confirmed and maintained through the treatment of others during destructive sexual contacts. One informant described:

*“It became a vicious loop that was hard to get out of. It also created the image of myself as ‘the slut’*,* so I somehow identified myself with the sex as well. If I wasn’t someone who let guys ‘use’ me*,* then who am I?”* (Woman, aged 28, No. 182).

Among those who ceased SASI, the core beliefs were changed in terms of stronger self-esteem and perception of self-worth: *“I have realized that I am worth something as a human being and I’m not a fuck doll”* (Woman, aged 27, No. 80). Some informants expressed an increased empathy for themselves as an example of this process: “*Liking oneself is very important. It helped me a lot”* (Woman, aged 22, No. 125).

The process of revision was described by some as taking place through *new experiences* and by others through *cognitive restructuring.* New experiences provided information that contradicted previous beliefs. One informant wrote:

*“I met my current boyfriend[…]. He questioned my sexual approaches when he saw that I didn’t want to.[.]. He himself has interrupted several times when he noticed that I was not committed and encouraged me to speak up when I do not want to*.*”* (Woman, aged 28, No. 180).

Initially, the informant acted based on previous beliefs about herself and others, but through the partner’s response she received information that contrasted with previous experiences. Another informant describes how the treatment from others was of great importance to the process: *“I feel that I have other values than sex. [//] I have received ‘confirmation’ of other things*,* such as that I have studied with good results”* (Woman, aged 31, No. 60).

If revision via experiences can be described as a movement from action to thought or outside in, then revision via cognitive restructuring is rather a process that moves from the inside out via changed thought patterns. Informants describe having been given the space and opportunity to reflect, question and re-evaluate their view of themselves: *“Long therapy with the world’s best therapist. I gained a sense of worth and care for myself. I don’t want to hurt myself anymore”* (Woman, aged 35, No. 46).

### Examples of new behaviors after revised core beliefs about the self

Within the theme “Revised core beliefs about the self” it is apparent how the informants’ changed self-image has become an important prerequisite for cessation of SASI. Examples of alternative behaviors that can replace self-injury related to the theme are about valuing and responding to oneself in more adequate and constructive ways. The following quote is an example of this: *“Nowadays I try to stay in the feeling and reformulate the self-hatred into something else. What’s hard isn’t about me and I don’t have to self-hate because I’m in a difficult event or experiencing difficult feelings”* (Woman, aged 27, No. 84). Other informants wrote: *“Comfort myself*,* pep myself up”* (Woman, aged 20, No. 193). And *“I started to give myself intrinsic value. I began to love myself. Then I stopped offending myself through others.”* (Woman, aged 27, No. 84).

### Evolved emotional competence

Informants described how, during the time of SASI, they had not been able to regulate intense emotions, but perceived and interpreted emotions as incomprehensible, unacceptable and unmanageable. SASI was often used to reduce these feelings, such as intense anxiety. For example, the lack of emotional competence is described like this: *“I couldn’t find a way out. I was in a vicious loop*,* I didn’t think I was worth more than that. They could treat me however they wanted*,* I just wanted to get away from my feelings”* (Woman, aged 25, No. 73).

Evolved abilities to identify, interpret and manage one’s own emotions in a more functional way were seen as helpful in the process towards cessation of SASI.

*“I have used therapy to process the most difficult feelings and occurrences I have experienced. On top of that*,* I have found ways to express what I’m carrying. I can write about it or talk about it with someone I trust. [//]. The more serious the problem is*,* the greater the importance of sharing it with someone. I also use methods and exercises on my own that I have learned in different forms of therapy. The basic insight is always to let the emotions come out and take up space. Never push them back”* (Man, aged 34, No. 149).

The process of developing the ability to regulate emotions more adequately was made through *understanding*, *acceptance*, and through *new coping skills.*

Informants described how it was helpful to gain insight into the function of emotions. Increased understanding of emotions made it possible to cope with them in other ways than through self-injuring behaviors. Informants described how it was helpful to be able to identify emotions, interpret emotions in new ways, reflect on them and put them into words. One informant wrote: *“Nowadays I try to think that they always pass [emotions]. I cry a lot. I’m trying to analyze them*,* reflect on them. When I was a teenager*,* I just tried to numb my feelings with everything I could”* (Woman, aged 25, No. 108).

Other informants described a greater ability to bear their feelings, to accept and make room for them, which reduced the need to self-injure. One informant wrote: *“I try not to be afraid to think about difficult things. I try to allow myself to grieve*,* cry or be angry”* (Woman, aged 30, No. 186).

Through increased understanding and acceptance of emotions, informants described having developed alternative ways to handle their emotions. These abilities reduced the need for self-injury, and SASI could be replaced by more constructive strategies. For example, some expressed that they had been helped to put their feelings into words and to remain in anxiety without acting self-destructively. One informant wrote: *“Nowadays I try to turn to friends*,* write it down*,* listen to music with lyrics that help me put my feelings into words*,* exercise. More rarely*,* I fall back into some form of self-destructive behavior”* (Woman, aged 32, No. 48).

### Examples of new behaviors after evolved emotional competence

The theme “Evolved emotional competence” reveals how informants had developed a better ability to cope with emotions and that this had reduced the need of SASI. The following quotes highlight examples of alternative behaviors linked to the theme: *“Try to accept that the feelings are there*,* but at the same time make sure that they do not get the better of you. Thinking about breathing. I take care of myself by*,* for example*,* taking care of basic needs such as sleep*,* food*,* hygiene*,* etc.”* (Woman, aged 20, No. 58). *“Now I have become friends with even the more difficult feelings*,* explore them through writing diaries*,* yoga and recovery”* (Woman, aged 26, No. 140). *“I use tools I received when I treated my PTSD*,* such as thinking that I won’t die from those feelings. It’s okay and it will pass”* (Woman, aged 28, No. 14).

### Increased relational competence

Informants described having experienced great loneliness and the only way to feel a sense of belonging was by offering their body for sex. Some emphasized a longing for closeness, love and relationship. They described casual sex as a way of trying to get that kind of affirmation. Others expressed not feeling worthy of more than violent sex on other people’s terms. In this regard, SASI may have been maintained because of relational needs that were not adequately met by others. One of the informants described:

*“I felt so bad about the betrayals from my family and was so lonely. No one could help me or comfort me when I felt bad*,* I didn’t know anyone. The only people I felt I had the right to hang out with were pigheaded men. No one else would have to put up with me. The fact that I can’t handle relationships allowed it to go on*,* and still allows it*,* even now that I don’t explicitly want to hurt myself. I don’t want to be abandoned*,* so it’s a safe way to give the body to someone. I also needed attention. And these men are everywhere*,* so it’s accessible to hurt oneself in this way”* (Woman, aged 27, No. 122).

Informants found it important to have new and contrasting relationships that better met the needs that SASI had previously compensated for, to be able to cease SASI. Examples of increased relational competence included the ability to enter a healthy relationship that gave positive affirmation and a sense of belonging. Relationships with partners, friends and relatives, including mutual affirmation in a constructive and lasting way, were highlighted as important contributions to stopping SASI:

*“That’s how I met the love of my life. Today we have been married for four years and have a little son who is four months old. It was my husband who made me stop. When I met him*,* I no longer needed what I needed then. He simply made me feel better”* (Woman, aged 33, No. 40).

Some informants highlighted parenthood as another important factor for the cessation of SASI:

*“The craving was always there*,* but later I let it go more or less completely when I had children and the thought of what might happen to them if it came out what their mother was doing. I want to be strong for my children. If I didn’t have kids*,* I don’t think I would have stopped.”* (Woman, aged 27, No. 114).

Increased relational competence was developed through *new relationship experiences* and/or through *new communication skills*. The informants described new ways of communicating with others, like confiding in others or asking for help. When they were met with a positive response and gained trust, it paved the way for a cessation of SASI.

### Examples of new behaviors after increased relational competence

The theme “Increased relationship competence” paints a picture of how the development of relational skills, experiences from new mutual and positive relationships and strengthened communication skills are highlighted as crucial for the cessation process of SASI. Alternative behaviors linked to this theme are largely about using one’s own network and being able to ask for and receive help and support from others. Informants described this in the following citations concerning alternative behaviors to cope with difficult feelings: *“When I feel bad now*,* I usually talk to my best friend*,* mother or my therapist”* (Woman, aged 27, No. 24). *“I talk to my husband*,* be with my son*,* talk to a person who I think can help”* (Woman, aged 29, No. 51). *“Talking to relatives and asking for help”* (Woman, aged 25, No. 73).

### Acquired meta-perspective

Informants emphasized that a perpetuating factor for SASI was the lack of insight concerning underlying functions of self-injurious behavior or the long-term negative consequences. SASI was reinforced by the anxiety-regulating, affirming or self-punishing effect and informants described being stuck in destructive patterns based on the short-term consequence:

*“That I felt so inadequate and worthless*,* that it was a ‘good way’ to both numb feelings*,* get validation and punish myself. It took a long time before I understood that my behavior was very destructive and that I was addicted to self-harm through sex”* (Woman, aged 26, No. 13).

The maintenance of SASI was sometimes described in terms of being stuck in vicious circles that were difficult to get out of: *“If someone hit me*,* I thought I deserved [it]. Afterwards*,* however*,* I got anxious about what I had done. Anxiety relief turned into anxiety”* (Woman, aged 26, No. 165).

The process related to the cessation of SASI when this maintenance occurred was *through insight and knowledge of SASI*. It was seen as helpful to better understand the causes of self-injurious behavior, both general and specific:

*“It was only about two years ago that I heard for the first time that there is such a thing*,* and that sex could be used as self-injury. This opened a whole new world for me and gave me an insight into myself*,* that maybe I’m not as worthless as I always thought because I had been unfaithful! An incredibly difficult feeling to live with*,* but with the information that this ‘condition’ exists actually made it all easier.”* (Woman, aged 33, No.2).

It was seen as helpful to increase the understanding of the mechanisms that maintained the behavioral pattern, which was in some cases done by getting sight of the conflict between the short-term benefit and the long-term negative consequences, and furthermore by realizing that the short-term benefit reinforced the negative loop. One informant wrote:

*“I started having a lot of panic attacks and generally feeling bad*,* so a friend made me seek help and it was only six months into therapy that I understood what I had been doing and why. I have understood that it is bad for me and know that my well-being in the long run will be worse from this self-injurious behavior*,* even if it eases things in the moment.”* (Woman, aged 24, No. 146).

Achieving a meta-perspective of SASI was seen as essential in the process of ceasing the behavior: *“I didn’t really know I had sex as self-injury until my counselor helped me put it into words and gain insight*,* so the help she gave me is invaluable.”* (Woman, aged 20, No. 194).

### Examples of new behaviors after acquired meta-perspective

The theme “Acquired meta-perspective” highlights how an increased understanding of the function and consequences of one’s own self-injury made it possible to exit the negative loop of the behavior and cease SASI. Alternative behaviors which were exemplified on this theme were: *“Now I have a crisis list to follow and usually I do crafts*,* drink tea or watch movies when the impulses come”* (Woman, aged 23, No. 3). *“I went to CBT therapy because I self-injured with razor blades*,* and I used the same way when I stopped hurting myself with sex*,* you learn good tricks there”* (Woman, aged 25, No. 108). *“Reflect*,* make chains*,* talk to someone I trust*,* write*,* fantasize about how I hurt myself and then let go of the imagination and what is difficult”* (Woman, aged 25, No. 117).

### Strengthened psychological empowerment

Informants who had ceased SASI described how they had developed psychological empowerment, a subjective perceived control over themselves and their situation. This included agency and increased self-determination, as well as the ability to shift guilt and responsibility from themselves. Some highlighted a political awareness and a norm-critical approach as a prerequisite for increased ego-strength. One wrote: *“I became healthier mentally. But I also became a more aware/active feminist and learned through that*,* that I and no one else has the right to my body. I started having sex for my own pleasure and desire”* (Woman, aged 20, No. 58).

Processes that strengthened the informants’ psychological empowerment were seen to occur through a *new relationship to the own body and sexuality*, as the following quote exemplifies:

*“After I started working in the Armed Forces*,* I also realized that sometimes I only have my own body to rely on. It didn’t matter if I hadn’t had sex in a long time because it carried me mile after mile anyway. It was good enough as it was*,* even though no one touched it. It showed me that it trusts me and then I realized that I have to trust it back. That we are a team”* (Woman, aged 20, No. 194).

Other informants reported that conquering the right to one’s sexuality and one’s pleasure was important to cease SASI. One informant put it this way:

*“I started working on my self-esteem and stopped hanging out with people who took advantage of me. I learned that sex should be something you do because you enjoy it. Sure*,* it can be brutal*,* dirty*,* and violent if you like it. But it should be something you like*,* enjoy. And above all*,* with a person you like and want to have sex with. Not just someone you do it with because it’s too hard to say no”* (Woman, aged 24, No. 30).

Understanding one’s right to set boundaries and thereby express *self-assertive behaviors* was another process that was seen in psychological empowerment. One informant wrote: *“I learned to say no and then I grew. Today I can better set boundaries and become stronger by denying someone my body”* (Woman, aged 40, No. 167).

Other expressions of self-assertive behaviors were exemplified by actively reducing areas where SASI could occur, i.e. preventing what could trigger or enable SASI. Informants said that they had stopped drinking alcohol, avoided club environments or chosen to stay away from the internet: *“I stopped using computers at all for a period of time to not fall back into old patterns. I’m not on internet sites where I’m at risk of being picked up”* (Woman, aged 31, No. 60). An informant describes how she completely stopped seeing men to reduce the risk of SASI:

*“The big part for me was when I became a lesbian. Even though I slept around with women*,* I felt so much better and didn’t get the same power differences as with men. I stopped hanging out in sex-positive circles*,* where BDSM was seen as sacred and cool”* (Woman, aged 19, No. 133).

Another process that increased empowerment was the *ability to transfer the responsibility* for the violence from oneself to the perpetrator(s), both in the present but also to those who had previously subjected them to incest, abuse or rape: *“I realized that I did not deserve to be hurt*,* and found a rage around the realization that men chose to hurt me just because I allowed it”* (Woman, aged 28, No 63).

Informants expressed how SASI had been able to arise and continue due to prevailing norms about gender roles, patriarchal structures and objectification of women’s bodies: *“I think it was because I learned as a child that men take what they want*,* when they want*,* no matter what you say… it just hurts a little less to say yes”* (Woman, aged 46, No. 159).

### Examples of new behaviors after strengthened psychological empowerment

The theme “Strengthened psychological empowerment” portrays how empowerment– through a *new relationship to one’s own body and sexuality*, via *self-assertive behaviors*, via the ability to *transfer responsibility* and via a *norm-critical perspective*– has contributed to a movement towards ceasing SASI. The following answer to the question “What do you usually do to deal with difficult feelings and events?” exemplifies alternative behaviors linked to the theme: *“Write a list of everything I feel guilty about and go through them and do my best to convince myself that none of them are my fault”* (Woman, aged 15, No. 177), *“Today I usually channel them into my feminist commitment”* (Woman, aged 28, No. 63) and *“Talk to the mirror and pretend I’m speaking up”* (Woman, aged 26, No. 165).

## Discussion

The aim of this study was to explore which psychological processes and abilities made it possible to break the maintenance cycle of SASI and thereby paved the way for the cessation of the behavior. The study found five abilities with twelve underlying psychological processes for ceasing SASI. The main findings of the study will be discussed further below.

A connection between the informants’ core assumptions, i.e. their subjective truth about themselves, and SASI, was found in the study. Informants who had ceased SASI had developed a more positive self-image and described expectations of treatment from others that were in line with this new image, for example that they deserved consideration and respect. Thus, vicious circles were created. A meta-analysis about cessation of NSSI shows that the factors of self-esteem, self-confidence, and hope, along with cognitive reappraisal, are particularly crucial for ceasing self-injury [15]. These results seem to be consistent with what has been found regarding the importance of revised core assumptions for ceasing SASI in this study.

The most common function of self-injury, both for NSSI and SASI, is emotion regulation [10, 20]. This study found that emotional competence was a skill that enabled the cessation of SASI. Competence was described as comprising an increased understanding of, acceptance of, and ability to manage one’s own feelings. The fact that emotional competence contributes to the cessation of SASI is consistent with the findings regarding NSSI. One’s own perception of the capacity to down-regulate emotions in the face of adversity is a key factor in discontinuing NSSI [11]. Poorer ability to regulate emotions is a factor that predicts ongoing self-injury [11]. An increased ability to accept emotions, an increased capacity for cognitive restructuring, and increased resilience are essential for an improved capacity for emotional regulation [15]. These aspects are like the psychological processes that were found regarding emotional competence in this study.

In this study it was described how close relationships with partners, friends, family or pets had been crucial for the cessation of SASI. Increased relational competence was found as a skill that paved the way for cessation. The ability seemed to have come about partly through new relationship experiences and partly through new communication skills. In a study where the role of social support in relation to self-injury was examined, the results showed that social support is associated with the reduction of NSSI and that the degree of perceived social support correlates with the degree of self-injurious behaviors. The study also found that hopefulness and resilience were mediating factors [22]. Engaging in relationships that bring emotional connection and create meaningfulness promotes motivation and strengthens the desire to stop self-injury, which is a key factor when it comes to ending NSSI [15]. This research can be related to what has emerged in this study. For example, new relational experiences gave the informants contact with emotional attachment that creates meaningfulness and new communication skills that made it possible to ask for and receive social support.

Informants described how, by becoming aware of patriarchal structures, gender roles, power differences and norms, they had been strengthened to break patterns and start thinking and acting in new ways. An observation was that the language that some of the informants used about the self-injurious behavior sometimes shifted the focus away from the perpetrator’s perspective and tended to make the men’s violence and sexual abuse invisible. Blaming victims of violence, so-called “secondary victimization”, is a reason why few people report sexual crimes. The phenomenon arises when victims of violence are met negatively or questioningly when they talk about what they have been through [1]. These tendencies were like those discussed in a study by Coates and Wade [4] where the authors, by examining legal judgments, demonstrated how, for example, the judicial system reflected and produced society’s norms and values through word choices that in various ways shrouded and diminished violence and exposure to violence. It became evident that the language used in violence cases concealed violence, concealed and mitigated the responsibility of the perpetrators, concealed the resistance of the victims, and pathologized and blamed the victims [4]. In this study, it was expressed that a conquered awareness of the right to one’s body and sexuality strengthened the individual’s psychological empowerment. This led to a shift in the discourse, which confirms the importance of word choice and attitudes. Acquiring knowledge about patriarchal structures revealed the perpetrator’s responsibility, which informants described as an important part of their process towards ending SASI, because the feminist value or opinion was too contrasting with the behavior.

Another sign of secondary victimization was comments about refraining from telling therapists or others about SASI because of a fear of being judged. This highlights the importance of therapists having a respectful and non-judgmental approach and helping individuals with SASI to place guilt and shame where it belongs [6]. Informants described how, during the time they used SASI, they were not aware of the function that maintained the self-injuring behavior. Some described getting information about the phenomenon as an eye-opener. Informants expressed feelings of relief and validation when they gained knowledge and encountered the concept of SASI, giving them a meta-perspective on their self-injury. In addition to relieving guilt, the insight also seems to reduce the shame caused by not being able to stop a behavior that seemed illogical and that they had perceived as destructive and unwanted but had not been able to end. The informants’ statements confirm the importance of increased awareness of SASI, both in society and on an individual level. If the phenomenon and the concept of SASI become accepted and highlighted, stigmatization and unreported cases can hopefully be reduced. The fact that people with experience of SASI have highlighted the importance of getting a name for the behavior, gaining access to existing knowledge that has created increased comprehensibility, and being treated with respect, empathy and professionalism is highlighted in previous research [6]. Professionals who possess knowledge of SASI can thus contribute to making the phenomenon visible and reducing taboos, and in parallel work to support clients in increasing their understanding of themselves by facilitating a meta-perspective around SASI.

Previous research describes the complexity that characterizes SASI when it comes to the motives and functions that prompt the self-injurious behavior [8], which also appears in this study. Different maintenance factors existed in parallel and SASI could fulfill different functions for one and the same person. Sometimes SASI was described as adding something such as closeness, proof of value, confirmed self-hatred or desired physical pain which was perceived as punitive or gave a sense of control. SASI was described at other times as reducing unwanted experiences such as anxiety, removing feelings of loneliness or providing a way to escape punishment and anger from partners. Several of these functions were described as co-varying and occurring in parallel for the same informant. This phenomenon has previously been described in relation to NSSI, where it has been found that one and the same self-injurious behavior can have multiple functions [20]. From a treatment perspective, this may become relevant. For example, for a therapeutic intervention to be helpful, it needs to be based on a careful assessment of what prompts and maintains SASI. What emerged in the results regarding the importance of the informant’s own understanding of their self-injury behavior under the theme “Meta-perspective on SASI” highlights the value of a functional perspective. A detailed analysis of SASI’s functions can clarify which alternative behaviors can replace self-injury or stop the urge to self-injure arising, i.e. make it possible to discontinue the maintenance of SASI. Based on this, a careful functional analysis appears to be one of the most important prerequisites for a change towards ceasing SASI.

### Strengths and limitations of the study

This study has some strengths and limitations. One limitation was the use of a questionnaire instead of interviews, which made it impossible to ask follow-up questions, which normally enrich and nuance the results and reduce the risk of misinterpretations. On the other hand, a questionnaire-based study made it possible to reach a large and broad group of informants that included individuals with both ongoing self-injury and previous behavior of SASI. The range of informants made it possible to discern a wider range of psychological processes and abilities and provided a rich and nuanced foundation.

Another limitation regarded the collection of data through the websites of organizations such as women’s shelters for women and girls. This resulted in a population mostly made up of women, hence, more research concerning men or persons with non-binary identification is needed in regard ceasing of SASI but also concerning SASI generally since the research is spars. Further, one possible selection bias might be an overestimation of particapants having a history of violence since women’s shelter especially address people in violent relationships.

A further issue that could be interesting to study is a comparison of processes and abilities needed for leaving NSSI compard to SASI since the behaviors are topographically different but similar in its underlying functions.

## Conclusions and clinical implications

In conclusion, psychological abilities that made it possible to cease SASI could be summarized in revised core beliefs about the self, evolved emotional competence, increased relational competence, acquired meta-perspective and strengthened psychological empowerment. The informants exemplified new behaviors they performed when they renounced SASI. These were described in the study as possible alternative behaviors which the acquired abilities generated. Overall, this study indicated aspects that could be important to promote, strengthen and develop to enable the cessation of SASI. With some caution, the results provide ideas about how the information could be used in clinical and therapeutic work with the target group. Hopefully, the results of the study can provide input on which psychological processes could form the focus during treatment. What the informants described as helpful in the process of stopping self-injury, as well as what support and help they wanted or found beneficial. For continued clinical research that can increase the understanding of effective treatment mechanisms for ceasing SASI the authors suggest that the following interventions, based on the results of this study, are tested and evaluated:

- Support clients in challenging negative core assumptions about themselves and strengthen self-esteem and experience of self-worth. Contribute conditions for questioning and revising dysfunctional assumptions and nuanced self-image, for example via methods such as cognitive restructuring.
- Present interventions that aim to strengthen clients’ emotional regulation. For example, contribute to increased understanding of, acceptance of, and ability to manage strong feelings, through psychoeducation about the function and duration of emotions.
- Highlight and pay attention to clients’ relational skills and support the development of communication skills. Focus on the importance of mutual relationships and of social support.
- Through increased knowledge and psychoeducation, contribute to clients acquiring meta-perspectives on self-injury and gaining insights into short- and long-term consequences. Support clients in exploring and understanding background factors and perpetuating their self-harming behaviors. Also have a focus on what can discontinue the maintenance based on the experiences of others and through the individually based conceptualization and functional analysis for the client.
- Encourage clients to recognize their right to set boundaries and increase their ability to practice assertive behaviors through skills training. Support clients in the process of changing the image of their own body and sexuality through conversations about consent and sexual health and rights. Help clients to place guilt and responsibility on perpetrators and open them up to the possibility of questioning limiting norms, challenging stereotypical gender roles and seeing through patriarchal structures.
- Contribute suggestions and curious exploration of potential alternative behaviors that could replace SASI, meet the client’s needs and constitute adaptive coping strategies.

## Electronic supplementary material

Below is the link to the electronic supplementary material.

Supplementary Material 1

## Data Availability

The datasets used and/or analysed during the current study available from the corresponding author on reasonablerequest.

## Abbreviations

ADHD/ADD: Attention deficit hyperactivity disorder/attention deficit disorder
CBT: Cognitive behavioral therapy
NGO: Nongovernmental organizations
NSSI: Nonsuicidal self–injury
PTSD: Post–traumatic stress disorder
SASI: Sex as self–injury
TA: Thematic analysis
