# Supplementary material for: Psychological processes and abilities for ceasing sex as self-injury– a qualitative study
Source: BMC Psychiatry. 2025 Jul 1;25:645. doi: 10.1186/s12888-025-07029-2 (PMC12217359; doi:10.1186/s12888-025-07029-2)
Supplement: Supplementary file 1 — Supplementary Material 1 [file 12888_2025_7029_MOESM1_ESM.docx]

| Appendix  Heading of the questionnaire:  This is a survey aimed at you who are over 15 years old and have or have had experiences of sex as self-injury. By sex as self-injury, we mean; That you have repeatedly sought out sexual situations that have caused you physical and/or psychological harm and that has affected you in your life. The survey is completely anonymous, and you can interrupt at any time. All you write will be anonymized so that it cannot be linked to any person or situation.  The questionnaire used for the study: |
| --- |
| 1. How old are you? |
| 1. What is your gender identity? E.g. woman, man, non-binary? |
| 1. What do you usually do to cope with negative feelings or occurrences? |
| 1. Describe your experiences of sex as self-injury |
| 4a. In which way did you have sex as self-injury? |
| 4b. How did it start? |
| 4c. How old were you when you had sex as self-injury? Do you still have it? |
| 4d. What made it continue? |
| 4e. Describe a typical occasion when you had sex as self-injury, what happened? |
| 4f. Think of a typical occasion. If this happened with another person, what relationship did you have to the other person and what was his/her age and gender? |
| 4g. If you have stopped, what made you stop? |
| 1. What experiences did you have of help and support when you had sex as self-injury? |
| 1. What did you want in terms of help, support and treatment from healthcare or other organizations working with help and support when you had sex as self-injury? |
